# Baseline entomologic data on malaria transmission in prelude to an indoor residual spraying intervention in the regions of Alibori and Donga, Northern Benin, West Africa

**DOI:** 10.1186/s12936-018-2507-y

**Published:** 2018-10-29

**Authors:** Albert S. Salako, Idelphonse Ahogni, Casimir Kpanou, Arthur Sovi, Roseric Azondekon, André A. Sominahouin, Filémon Tokponnon, Virgile Gnanguenon, Fortuné Dagnon, Laurent Iyikirenga, Martin C. Akogbeto

**Affiliations:** 1 Centre de Recherche entomologique de Cotonou (CREC), Cotonou, Benin; 2 Faculté des Sciences et Techniques de l’Université d’Abomey-Calavi, Abomey-Calavi, Benin; 3 PMI VectorLink Project, Abt Associates, Bamako, Mali; 4 University of Wisconsin Milwaukee, Milwaukee, WI USA; 5 Faculté des Sciences Humaines et Sociales de l’Université d’Abomey-Calavi, Abomey-Calavi, Benin; 6 Programme Nationale de Lutte contre le Paludisme, Cotonou, Benin; 7 PMI VectorLink Project, Abt Associates, Bujumbura, Burundi; 8 US President’s Malaria Initiative, US Agency for International Development, Cotonou, Benin; 9 PMI VectorLink Project, Abt Associates, Cotonou, Benin

**Keywords:** Malaria transmission, *Anopheles gambiae* s.l., IRS, Alibori, Donga, Benin

## Abstract

**Background:**

Despite the success of indoor residual insecticide spraying (IRS) in Africa, particularly in Benin, some gaps of information need to be filled to optimize the effectiveness of this intervention in the perspective of the country’s effort to eliminate malaria. In anticipation to the 2018 IRS campaign in two targeted regions of northern Benin, this study aimed, to collect baseline information on vector composition, spatio-temporal variation and peak malaria transmission in the Alibori and Donga, two targeted regions of northern Benin. Information collected will help to better plan the implementation and later on the impact assessment of this IRS campaign.

**Methods:**

The study was carried out in four districts of the two IRS targeted regions of northern Benin. Human landing catches and pyrethrum spray catches protocols were used to assess the biting rate (HBR) and, biting/resting behaviour of malaria vector populations. After morphological identification of collected *Anopheles*, the heads and thoraxes of *Anopheles gambiae* sensu lato (s.l.) were analysed by the ELISA CSP tests to estimate the sporozoite index (SI). The entomological inoculation rate was calculated as the product of mosquito biting rate (HBR) and the SI.

**Results:**

The biting rates of *An. gambiae* s.l., the major vector in this study sites, varied significantly from region to region. It was higher: in rural than in urban areas, in rainy season than in dry season, indoors than outdoors. Overall, SI was comparable between sites. The highest EIRs were observed in the Donga region (16.84 infectious bites/man/month in Djougou district and 17.64 infectious bites/man/month in Copargo district) and the lowest in the Alibori region (10.74 infectious bites/man/month at Kandi district and 11.04 infectious bites/man/month at Gogounou district).

**Conclusion:**

This study showed the heterogeneous and various nature of malaria epidemiology in Northern Benin. Indeed, the epidemiological profile of malaria transmission in the Alibori and Donga regions is made of a single season of transmission interrupted by a dry season. This period of transmission is relatively longer in Donga region than in Alibori. This information can be used to guide the extension of IRS in the Alibori and in the Donga, by primarily targeting areas with short periods of transmission, and easy to cover.

## Background

Indoor residual spraying (IRS) and insecticide-treated nets (ITNs) are two key and effective strategies designed to interrupt malaria transmission [1–3]. IRS has greatly contributed to reduce or eliminate malaria from many areas of the world, particularly in situations where mosquito vectors feed and rest indoors and where the transmission of malaria is seasonal [4–7]. In Benin, after 6 years of intervention, IRS has proved to be an effective vector control intervention [8]. Started in 2008 in the Oueme region (southern Benin), then relocated to the Atacora region (North Benin) from 2011 to 2015, the intervention was effective in reducing the level of malaria transmission [8–10]. The same trend has been observed in other sub-Saharan countries with this intervention: Swaziland, Botswana, South Africa, Zimbabwe and Mozambique [11], Madagascar [12], Equatorial Guinea (Bioko Island) [13–15], in Uganda [16], Kenya [17] and Tanzania [18]. Unfortunately, IRS effectiveness is being jeopardized by the spread and intensification of insecticide resistance, including to pyrethroids [19–24] and more recently to bendiocarb [25–27]. Density and distribution of *Anopheles,* vectors of malaria vary according to the region and the time of year, and these variations can modify malaria transmission levels [28–31]. Several studies have shown that malaria infection is influenced by environmental factors, such as temperature, precipitation, and relative humidity that vary from region to region [32]. However, in most parts of Africa, there are still gaps in information regarding the dynamics of malaria transmission resulting in the implementation of vector control interventions without sufficient decision-making basis [33–35].

This was the case of Benin where, from 2008 to 2009, a single round of IRS instead of two was implemented in the Oueme region to cover the period of malaria transmission [9]. In 2017, the IRS campaign, with pirimiphos methyl (Actellic 300CS), has targeted all eligible households in the Alibori and Donga regions. These two regions being located in two different eco-geographical areas despite their proximity, it was hypothesized that variations in vectors ecology may affect the micro-epidemiology of malaria. It is in this context that this study was initiated with the aim of obtaining useful information for a better planning and assessment of IRS intervention.

## Methods

### Study site

**Fig. 1:**
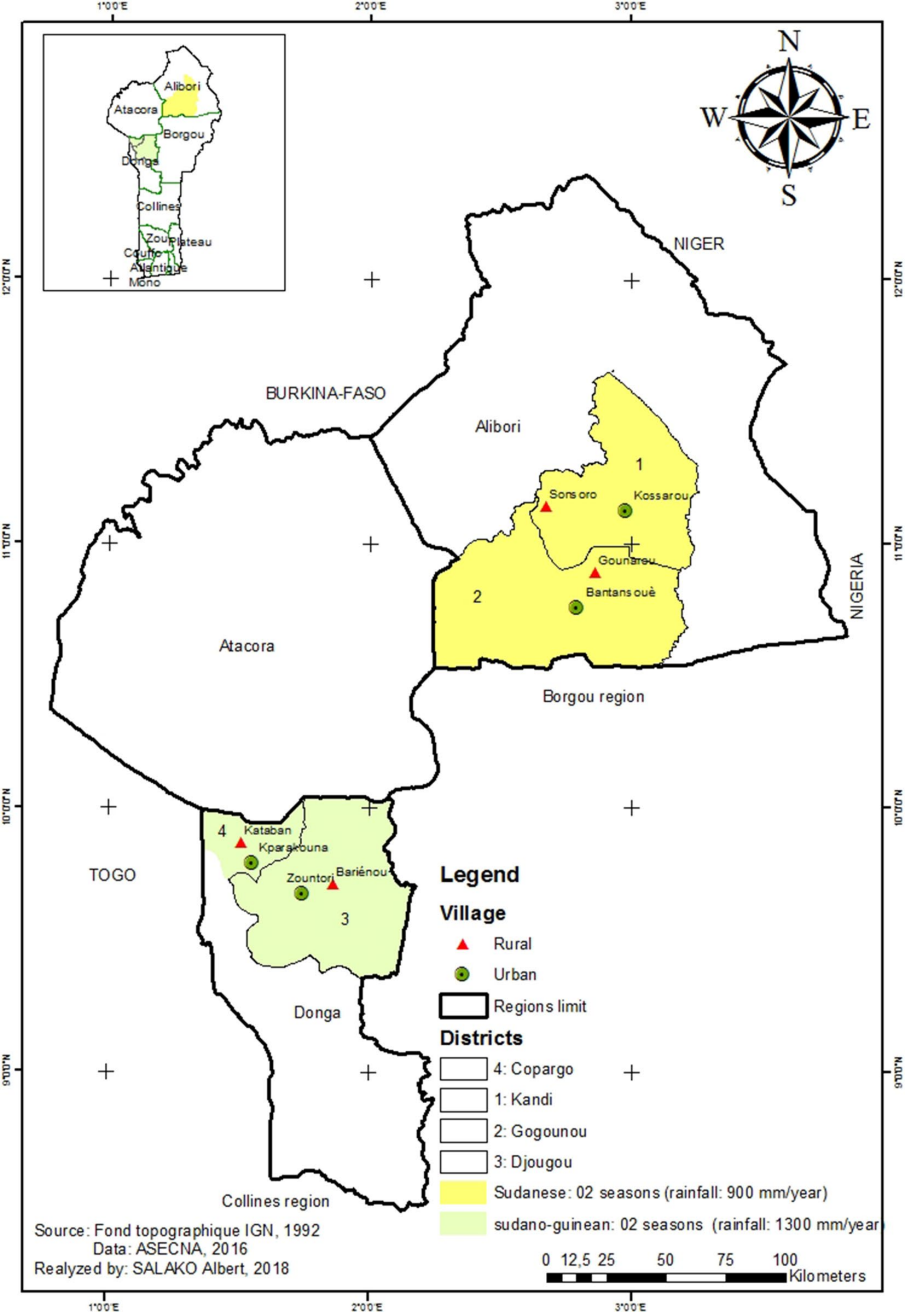
Study area and mosquito collections sites in Northern Benin

Entomological data were collected from two regions with two districts each: Alibori (Gogounou, Kandi) and Donga (Copargo, Djougou) (Fig. 1). The region of Alibori is characterized by a Sudanese climate and the Donga by a Sudano-guinean climate, with a single dry season (December to May) and a single rainy season (June to November). The annual average rainfall varies between 700–1200 mm and 1200–1300 mm, respectively in Alibori and Donga regions. The average monthly temperature varies between 23 and 40 °C. The region of Donga has more rivers than the region of Alibori. The major economic activity is farming of cotton, maize and millet [36, 37].

Malaria prevalence is generally higher in Donga region than in Alibori [38]. Long-lasting insecticide-treated mosquito nets (LLINs) distributed every 3 years throughout the mass campaign distribution are the main tools used to prevent human–vector contact in the four districts. In each district, two sites, one in urban and one in rural setting, were selected for mosquito collections. These sites are:

- In Djougou district:
  Zountori: a urban area of Djougou (09°42′10.1″ N latitude and 01°40′55.4″ E longitude);
  Barienou: a rural village of Djougou (09°42′58.4″ N latitude and 01°46′06.5″ E longitude) (Fig. 1).
- In Copargo district:
  Parakounan: a urban area of Copargo (09°50′19.3″ N latitude and 01°32′39.5″ E longitude);
  Kataban: a rural village of Copargo (09°54′34.3″ N latitude and 01°31′26.9″ E longitude);
- In Kandi district:
  Kossarou: a urban area of Kandi (11°07′29.32″ N latitude and 2°56′9.57″ E longitude);
  Sonsoro: a rural village of Kandi (11°4′58.91″ North latitude and 2°13′37.60″ East longitude);
- In Gogounou district:
  Bantansoue: a urban area of Gogounou (10°50′30.6″ N latitude and 2°50′20.3″ E longitude);
  Gounarou: a rural village of Gogounou (10°52′20.8″ N latitude and 2°50′51.2″ E longitude) (Fig. 1).

Sampling of *Anopheles* vectors of malaria was conducted from May 2016 to February 2017.

### Ethical considerations

The protocol of this study has been reviewed and approved by the Institutional Ethics Committee of the Center for the Research in Entomology of Cotonou (IECC). Verbal consent was obtained from local mosquito collectors before being involved in the study. They subsequently received a vaccine against yellow fever as a prophylactic measure. An agreement with health facilities close to sites was also obtained for the free anti-malarial treatment of mosquito collectors who would suffered from malaria.

### Anopheles adult collection

Malaria transmission dynamics were assessed in the 08 identified and described sites in the study area. This is why two classical methods were used for the sampling of *Anopheles* mosquitoes. The first sampling method is termed as Human Landing Catch (HLC), and was carried out from 9:00 p.m. to 5:00 a.m. in 2 nights per month per district, enabled the evaluation of the frequency of human–vector contact (human biting rate). For this purpose, four volunteers (02 inside and 02 outside) captured mosquitoes in 02 randomly selected houses per site, making a total of eight collectors per district per night. The collectors are rotated in the different houses to avoid biases related to their ability or their individual attractiveness. The second sampling method is pyrethrum spray catch (PSC) which was carried out through two morning sessions (between 6 a.m. and 8:30 a.m.) per month in 40 houses per district. This method allowed the collection of resting mosquitoes inside houses.

### Mosquito processing

These mosquitoes as well as those caught on human bait were examined for *Plasmodium falciparum* infectivity. After each collection, collected mosquitoes were counted and morphologically identified using the taxonomic key of Gillies and De Meillon [39]. Females of *An. gambiae* s.l. (refered to as *An. gambiae* thereafter in the text) caught on human bait were then dissected to assess their physiological age [40]. Each specimen was finally stored in a labelled Eppendorf tube containing silicagel for further laboratory analysis.

The heads and thorax of females of *An. gambiae* collected at each site were analysed by enzyme-linked immunosorbent assay (ELISA) according to the method described by Wirtz et al. [41]. This allows the detection of *P. falciparum* infection and the calculation of infectivity rates.

### Estimation of entomological parameters

The *human biting rate* (HBR) for identified vector species was calculated as the number of *An. gambiae* caught per person per night of sampling effort. The *sporozoite index* (SI) is the proportion of *An. gambiae* s.l. with circumsporozoite protein of *P. falciparum*: SI = (Thorax +/Thorax analysed) × 100. The *parity rate* is a percentage of *An. gambiae* that have laid eggs at least once (parous) out of the total number of *An. gambiae* dissected for the examination of the physiological status of their ovaries. It indicates the proportion of older mosquitoes within the population during the survey. The *entomological inoculation rate* (EIR), a key variable expressing the malaria transmission level and which is defined as the number of infective bites received/man/night. It is the product of *An. gambiae* biting rate and SI data acquired from HLCs and the ELISA tests, respectively.

### Data analysis

Data were analysed with the R statistics software, version 2.8. The Poisson method was used to estimate the confidence intervals [42] of HBRs and EIRs of *An. gambiae.* The binomial method for calculation of confidence intervals [42] was used to estimate the confidence intervals of parity and infectivity rates of *An. gambiae.* The unconditional maximum likelihood estimation method Wald, or median unbiased estimation (mid-p) of the risk ratio (RR) followed by their confidence intervals obtained using the normal approximation or the exact method p-values obtained by Khi^2^ or mid-p.exact [43] was used to compare EIR. Proportion comparison tests were used to compare SI and parity rates. A difference is considered as significant when the p-value is less than 0.05.

## Results

### Vector species composition

A total of 3876 specimens of *Anopheles* mosquitoes from six different species were collected in the regions of Alibori and Donga. The most abundant species was *An. gambiae*, which accounted for 97.72% (3788/3876) of the collected vectors, followed by *Anopheles funestus* (1.88%, 73/3876). The biting behaviour of these two vector species was generally higher in the Donga compared to the Alibori. It should be noted that *An. funestus* was mainly collected in the district of Djougou which provided about 82.2% (60/73) of the specimens (Table 1). Among *Anopheles* species which were collected, there were only 9 *Anopheles coustani* (0.23%), 3 *Anopheles pharoensis* (0.077%), 2 *Anopheles ziemanni* (0.05%) and 1 *Anopheles paludis* (0.025%) (Table 1).

**Table 1:** Vector species composition in all districts, May 2016–February 2017

Regions	Districts	*Anopheles gambiae* s.l.	*Anopheles funestus*	*Anopheles pharoensis*	*Anopheles ziemanni*	*Anopheles coustani*	*Anopheles paludis*
Donga	Djougou	1086	60	0	0	1	0
	Copargo	1188	8	2	1	8	1
	Sub-total	2274	68	2	1	9	1
Alibori	Kandi	869	4	0	1	0	0
	Gogounou	645	1	1	0	0	0
	Sub-total	1514	5	1	1	0	0
	Total	3788	73	3	2	9	1

### Variability of human biting rates

The biting rate of *An. gambiae* varied significantly from region to region: 6.55–7.51 bites/man/night (b/m/n) in the Donga against 4.4–4.78 bites/man/night in the Alibori (p < 0.05) (Table 2). The HBR was four times higher in rural areas compared to urban areas (RR = 4.27; p **< **0.0001). The data also show that the average biting rate of *An. gambiae* was higher indoors (6.37 bites/man/night) than outdoors (5.25 bites/man/night). In the dry season, it was 2.27 bites/man/night compared to 9.95 bites/man/night in the rainy season, an increase of more than four times (RR = 4.38; p **< **0.0001) (Table 2). In both regions, the highest biting rates were observed between June and October with a peak in October and August, respectively in Alibori and Donga (Table 2).

**Table 2:** *An. gambiae* s.l. average monthly biting trends by district, settings and seasons (May 2016 to February 2017)

Variables	Modalities	May	June	July	August	October	January	February	HBR/night	CI-95%	RR and CI-95%	p (Wald)
Donga	Djougou	2	2.88	9.13	13.06	9.06	2.38	5.13	6.55^a^	[6.07–7.06]	1	–
	Copargo	0.75	7.06	10.56	16.63	10.5	1.5	2.25	7.51^b^	[7.00–8.06]	1.15 [1.03–1.27]	0.0089
Alibori	Kandi	0.13	4.19	6.75	8.75	10.81	0.25	0.31	4.78^c^	[4.37–5.22]	0.73 [0.65–0.82]	< 0.001
	Gogounou	0.38	1.31	4.56	9	10.63	2	0.94	4.4^c^	[4–4.83]	0.67 [0.59–0.76]	< 0.001
Settings	Urban area	0.13	0.63	2.78	5.09	4.28	0.72	0.78	2.18^a^	[1.99–2.39]	1	–
	Rural area	1.50	7.09	12.72	18.63	16.22	2.34	3.53	9.42^b^	[9.01–9.85]	4.27 [3.85–4.75]	< 0.0001
Location	Indoor	1.06	3.53	8.50	12.13	11.97	1.97	2.81	6.37^a^	[6.03–6.72]	1	–
	Outdoor	0.56	4.19	7.00	11.59	8.53	1.09	1.50	5.25^b^	[4.95–5.58]	0.83 [0.76–0.89]	< 0.001
Seasonality	Dry season	0.81	–	–	–	–	1.53	2.16	2.27^a^	[2.07–2.47]	1	–
	Rainy season	–	3.86	7.75	11.86	10.25	–	–	9.95^b^	[9.51–10.40]	4.38 [3.97–4.83]	< 0.0001

^a,b,c^The values of the same variable with different letters are statistically different

### Sporozoite index of *Anopheles gambiae*

Overall, out of a total of 3788 head-thoraxes of *An. gambiae* assessed by ELISA, approximately 305 were found to be positive for the circumsporozoitic antigen of *P. falciparum*, which equates to a mean SI of 8.05% [7.20–8.96]. The SI were similar between urban (6.35% [4.63–8.46]) and rural (8.42% [7.6–9.45]) areas (p = 0.086). The highest infection rates were observed in Gounarou (11.65% [8.48–15.46]) and Kossarou (11.36% [5.58–19.90]) and the lowest in Bantansoue (4.44% [2.38–7.46]) (Table 3).

**Table 3:** Sporozoite index of *Anopheles gambiae* s.l.

Districts	Sites	May	June	July	August	October	January	February	Mean	Mean	Mean (7 months)	CI 95%	p-value
		(DS)	(RS)	(RS)	(RS)	(RS)	(DS)	(DS)	(DS)	(RS)			
Kandi	Sonsoro*												
	Thorax	38	119	175	166	244	10	29	77	704	781		0.212
	Thorax+	6	6	9	4	30	0	0	6	49	55		
	SI	15.79	5.04	5.14	2.41	12.30	0	0	7.79^a^	6. 96^a^	7.042	[5.34–9.06]	
	Kossarou												
	Thorax	7	4	6	16	49	2	4	13	75	88		
	Thorax+	1	0	0	0	8	1	0	2	8	10		
	SI	14.29	0	0	0	16.33	50	0	15.38^a^	10.67^a^	11.36	[5.58–19.90]	
	Total												
	Thorax	45	123	181	182	293	12	33	90	779	869		
	Thorax+	7	6	9	4	38	1	0	8	57	65		
	SI	15.56	4.88	4.97	2.20	12.97	8.33	0	8.89^a^	7.32^a^	7.47	[5.81–9.43]	
Gogounou	Gounarou*												
	Thorax	7	8	33	93	167	22	22	51	301	352		0.0016
	Thorax+	0	1	2	10	27	0	1	1	40	41		
	SI	0	12.50	6.06	10.75	16.17	0	4.55	1.96^a^	13.29^b^	11.65	[8.48–15.46]	
	Bantansoue												
	Thorax	0	23	64	72	71	33	30	63	230	293		
	Thorax+	0	1	1	2	5	3	1	4	9	13		
	SI	–	4.35	1.56	2.78	7.04	9.09	3.33	6.35^a^	3.91^a^	4.44	[2.38–7.46]	
	Total												
	Thorax	7	31	97	165	238	55	52	114	531	645		
	Thorax+	0	2	3	12	32	3	2	5	49	54		
	SI	0	6.45	3.09	7.27	13.45	5.45	3.85	4.39^a^	9.23^a^	8.37	[6.35–10.78]	
Total (Alibori region)	Thorax	52	154	278	347	531	67	85	204	1310	1514		
	Thorax+	7	8	12	16	70	4	2	13	106	119		
	SI	13.46	5.19	4.32	4.61	13.18	5.97	2.35	6.37^a^	8.09^a^	7.86	[6.55–9.33]	
Djougou	Barienou*												
	Thorax	56	57	251	198	145	51	171	278	651	929		0.223
	Thorax+	15	4	6	25	16	3	15	33	51	84		
	SI	26.79	7.02	2.39	12.63	11.03	5.88	8.77	11.87^a^	7.83^a^	9.04	[7.27–11.07]	
	Zountori												
	Thorax	0	0	33	59	41	18	6	24	133	157		
	Thorax+	0	0	1	5	2	1	0	1	8	9		
	SI	–	–	3.03	8.47	4.88	5.56	0	4.17^a^	6.02^a^	5.73	[2.65–10.60]	
	Total												
	Thorax	56	57	284	257	186	69	177	302	784	1086		
	Thorax+	15	4	7	30	18	4	15	34	59	93		
	SI	26.79	7.02	2.46	11.67	9.68	5.80	8.47	11.26^a^	7.53^a^	8.56	[6.96–10.38]	
Copargo	Kataban*												
	Thorax	13	189	274	284	198	35	56	104	945	1049		1
	Thorax+	0	8	16	34	21	3	0	3	79	82		
	SI	0	4.23	5.84	11.97	10.61	8.57	0	2.88^a^	8.36^a^	7.81	[6.26–9.61]	
	Kparakouna												
	Thorax	1	11	27	61	21	4	14	19	120	139		
	Thorax+	0	0	0	6	5	0	0	0	11	11		
	SI	0	0	0	9.84	23.81	0	0	0^a^	9.16^a^	7.91	[4.01–13.71]	
	Total												
	Thorax	14	200	301	345	219	39	70	123	1065	1188		
	Thorax+	0	8	16	40	26	3	0	3	90	93		
	SI	0	4	5.32	11.59	11.87	7.69	0	2.44^a^	8.45^b^	7.83	[6.36–9.50]	
Total (Donga region)	Thorax	70	257	585	602	405	108	247	425	1849	2274		
	Thorax+	15	12	23	70	44	7	15	37	149	186		
	SI	21.43	4.67	3.93	11.63	10.86	6.48	6.07	8.71^a^	8.06^a^	8.18	[7.08–9.38]	
Total (rural areas)	Thorax	114	373	733	741	754	118	278	510	2601	3111		0.086
	Thorax+	21	19	33	73	94	6	16	43	219	262		
	SI	18.42	5.09	4.50	9.85	12.47	5.08	5.76	8.43^a^	8.42^a^	8.42	[7.46–9.45]	
Total (urban areas)	Thorax	8	38	130	208	182	57	54	119	558	677		
	Thorax+	1	1	2	13	20	5	1	7	36	43		
	SI	12.50	2.63	1.54	6.25	10.99	8.77	1.85	5.88^a^	6.45^a^	6.35	[4.63–8.46]	
Grand total	Thorax	122	411	863	949	936	175	332	629	3159	3788		
	Thorax+	22	20	35	86	114	11	17	50	255	305		
	SI	18.03	4.87	4.06	9.06	12.18	6.29	5.12	7.95^a^	8.07^a^	8.05	[7.20–8.96]	

SI: sporozoite index; CI: confidence interval; *: rural area; DS: dry season; RS: rainy season

^a,b^The SI/season of the same site bearing different letters are statistically different

Overall, mosquito infectivity was 7.48% [5.81–9.43], 8.37% [6.35–10.78], 8.56% [6.96–10.38] and 7.82% [6.36–9.50], respectively in the districts of Kandi, Gogounou, Djougou and Copargo (Table 3). By cumulating data by region, SI was also similar (7.86% [6.55–9.33] in the Alibori versus 8.18% [7.08–9.38] in the Donga, p = 0.769) (Table 3).

Cumulative data from both regions show similar average of infectivity rates in the rainy season (8.07% [7.14–9.07]) and in the dry season (7.95% [5.95–10.34]) (p = 0.981). In Kandi, Gogounou and Djougou, mosquito infectivity was respectively 8.89% [3.91–16.76], 4.38% [1.43–9.93] and 11.26% [7.92–15.37] in the dry season versus 7.32% [5.58–9.37], 9.23% [6.90–12.01] and 7.53% [5.77–9.60] in the rainy season (p > 0.05). Conversely, in Copargo, the SI was significantly higher in the rainy season (8.45% [6.84–10.28]) than in the dry season (2.44% [0.63–7.50]) (p = 0.0297) (Table 3).

### Entomological inoculation rate of *Anopheles gambiae*

Tables 4 and 5 show the spatio-temporal variation in entomological inoculation rates (EIR). A variation of the EIR across districts was significantly higher in the rainy season (June, July, August and October) than in the dry season (May, January and February) (p < 0.05) (Table 4).

**Table 4:** Entomological inoculation rates (EIR) per district according to the season

Districts	Parameters	Dry season	Rainy season	Total	RR (95% CI)	p (Wald)
		(May–January–February)	(June–October)			
Kandi	HBR/night	0.25	7.625	4.78		
	SI	8.89	7.32	7.48		
	EIR/night	0.022	0.558	0.358		
	EIR/month	0.66^a^	16.74^b^	10.75	1	–
Gogounou	HBR/night	1.25	6.375	4.4		
	SI	4.4	9.2	8.37		
	EIR/night	0.055	0.588	0.368		
	EIR/month	1.645^a^	17.648^b^	11.06	1.03 [1.012–1.046]	0.0006
Djougou	HBR/night	3.4	8.53125	6.55		
	SI	11.3	7.5	8.56		
	EIR/night	0.38	0.64	0.561		
	EIR/month	11.48^a^	19.26^b^	16.85	1.56 [1.54–1.58]	< 0.001
Copargo	HBR/night	1.65	11.1875	7.5		
	SI	2.44	8.45	7.82		
	EIR/night	0.04	0.945	0.588		
	EIR/month	1.21^a^	28.36^b^	17.66	1.64 [1.62–1.66]	< 0.001

EIR: entomological inoculation rate; RR: rate ratio; CI: confidence interval; p-Wald: p-value of the significance of the ratio of entomological inoculation rate between districts

^a,b^The EIR/season of the same district bearing different letters are statistically different

**Table 5:** Monthly variation of EIR per site and per district

Districts	Sites	May	June	July	August	October	January	February	EIR/night	EIR/month	EIR/year	RR (95% IC)	P (Wald)
Kandi	Sonsoro^a^												
	HBR/night	0	8.25	13.25	15.75	17.63	0.38	0.38					< 0.0001
	EIR/night	0	0.42	0.68	0.38	2.17	0	0	0.6	18.08	216.96	1	
	Kossarou												
	HBR/night	0.25	0.13	0.25	1.75	4	0.13	0.25					
	EIR/night	0.04	0	0	0	0.65	0.06	0	0.12	3.47	41.7	0.19 [0.17–0.21]	
	Total												
	HBR/night	0.13	4.19	6.75	8.75	10.81	0.25	0.31					
	EIR/night	0.019	0.204	0.336	0.192	1.402	0.021	0	0.358	10.75	128.94		
Gogounou	Gounarou^a^												
	HBR/night	0.75	0.63	3	10.88	14.38	1.75	1					< 0.0001
	EIR/night	0	0.08	0.18	1.17	2.32	0	0.05	0.57	17.2	206.43	1	
	Bantansoue												
	HBR/night	0	2	6.13	7.13	6.88	2.25	0.88					
	EIR/night	0	0.09	0.1	0.2	0.48	0.2	0.03	0.17	5.17	62.05	0.30 [0.28–031]	
	Total												
	HBR/night	0.38	1.31	4.56	9	10.63	2	0.94					
	EIR/night	0	0.08	0.14	0.65	.43	0.11	0.04	0.369	11.06	132.73		
Djougou	Barienou^a^												
	HBR/night	4	5.75	15.88	20	14.38	4.38	9.75					< 0.0001
	EIR/night	1.07	0.4	0.38	2.53	1.59	0.26	0.86	1.003	30.1	361.19	1	
	Zountori												
	HBR/night	0	0	2.38	6.13	3.75	0.38	0.5					
	EIR/night	0	0	0.03	0.085	0.049	0.056	0	0.057	1.72	20.64	0.11 [0.10–0.12]	
	Total												
	HBR/night	2	2.88	9.13	13.06	9.06	2.38	5.13					
	EIR/night	0.54	0.20	0.22	1.52	0.88	0.14	0.43	0.562	16.85	202.17		
Copargo	Kataban^a^												
	HBR/night	1.25	13.75	18.75	27.88	18.5	2.88	3					< 0.0001
	EIR/night	0	0.582	1.095	3.337	1.962	0.246	0	1.027	30.81	369.72	1	
	Kparakouna												
	HBR/night	0.25	0.38	2.38	5.38	2.5	0.13	1.5					
	EIR/night	0	0	0	0.529	0.595	0	0	0.151	4.52	54.24	0.14 [0.13–0.15]	
	Total												
	HBR/night	0.75	7.06	10.56	16.63	10.50	1.50	2.25					
	EIR/night	0	0.28	0.56	1.92	1.24	0.11	0	0.589	17.66	211.91		
Urbanization	Rural areas^a^												
	HBR/night	1.5	7.09	12.72	18.63	16.2	2.34	3.53					< 0.0001
	EIR/night	0.27	0.36	0.57	1.83	2.023	0.119	0.203	0.793	23.79	285.54	1	
	Urban area												
	HBR/night	0.13	0.63	2.78	5.09	4.28	0.72	0.78					
	EIR/night	0.016	0.017	0.043	0.318	0.470	0.063	0.014	0.138	4.15	49.83	0.176 [0.174–0.179]	

EIR: entomological inoculation rate; RR: rate ratio; CI: confidence interval of RR; p-Wald: p-value of the significance of the ratio of entomological inoculation rate between the rural and urban areas

^a^Rural area

**Fig. 2:**
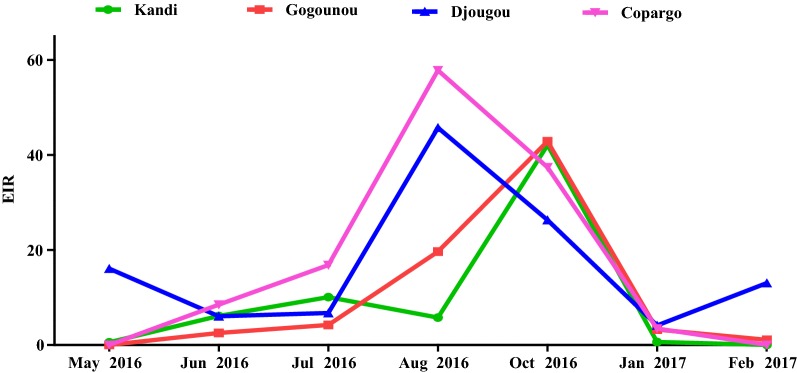
Monthly variation of EIR by district

Overall, the lowest infectivity was observed in Kandi (10.74 infective bites/man/month) and Gogounou (11.04 infective bites/man/month) in Alibori region than in Djougou (16.84 infective bites/man/month) and Copargo (17.64 infectious bites/man/month) in Donga region (Table 4). In the four districts, the infectivity was higher in rural areas than in urban areas (p < 0.05). Cumulative data revealed an average EIR of 23.79 infectious bites/man/month in rural areas versus 4.15 infectious bites/man/month in urban areas (p < 0.0001) (Table 5). In Alibori’s districts (Kandi and Gogounou), the period of malaria transmission was relatively shorter than in Donga’s districts where it extended from May to February at Djougou and, from June to January at Copargo (Fig. 2). In Alibori region, the peak of transmission was recorded in October at Kandi (42.06 infectious bites/man/month) and Gogounou (42.86 infectious bites/man/month). Conversely, in Donga region, it was observed in August at Djougou (45.6 infectious bites/man/month) and Copargo (57.6 infectious bites/man/month) (Fig. 2 and Table 5).

### Seasonal variation of the parity of *Anopheles gambiae*

Ovaries dissection of 1640 females of *An. gambiae* collected through HLCs were performed to determine parity rates. 1185 (72.3%) were found parous. Vectors’ parity rate was 81.90% [73.19–88.73], 91.38% [81.01–97.14] and 89.13% [76.43–96.37] during the dry season against 71.14% [66.39–75.56], 70.59% [65.90–74.96] and 68.58% [62.57–74.16] during the rainy season (p < 0.05), respectively in Djougou, Copargo and Gogounou. In Kandi, no significant difference was observed between parous rates of both seasons, probably due to the low number of mosquitoes dissected in the dry season (Table 6). Overall, cumulative data showed an average parous rate estimated at 86.70% [81.45–90.90] in the dry season against 70.04% [67.58–72.41] in the rainy season (p < 0.001).

**Table 6:** Parity rates of *Anopheles gambiae* s.l.

Districts	Dry season (May–January–February)			Rainy season (June–October)			p-value
	Number dissected	Parous	Parous rate (%)	Number dissected	Parous	Parous rate (%)	
Djougou	105	86	81.90	395	281	71.14	0.0361
Copargo	58	53	91.38	408	288	70.59	0.0014
Kandi	9	9	100	358	248	69.27	0.1055
Gogounou	46	41	89.13	261	179	68.58	0.0074
Total	218	189	86.70	1422	996	70.04	0.00042

## Discussion

The study of the dynamics of malaria transmission is a prerequisite to not only understand the epidemiology of this disease, but also to establish effective and targeted control of mosquito, vectors of diseases [44]. The entomological monitoring that we carried out revealed that *An. gambiae* and *An. funestus* were the main malaria vectors in Alibori and Donga regions, which confirms the results of previous studies conducted in West Africa, and specifically in Benin [45–47]. *Anopheles gambiae* appeared as the major vector (97.72%) in the two regions. This finding corroborates previous data published by Aikpon et al. [48] in Atacora and Gnanguenon et al. [47] in Kandi and Malanville, two districts of the region of Alibori. According to Akogbéto et al. [49], in Alibori and Donga regions, *An. gambiae* is composed of two sibling species (*An. gambiae* and *Anopheles coluzzii*) whose proportions vary depending on the region. In Alibori region, species composition showed *An. coluzzii (*62.2%) and *An. gambiae (*37.8%). In Donga, *An. gambiae* was the most abundant (64.7%). The two species were present throughout the transmission season [49]. *Anopheles gambiae* showed endophagic tendency. This behaviour of *An. gambiae* which feeds on man, preferentially inside houses, is justified by the fact that *An. gambiae* populations rest exclusively indoors during the rainy season, a period of vector abundance. However, this strong endophagy could also be facilitated by the alteration of the repulsive and lethal properties of the LLINs distributed in 2014 within the communities in both regions. In fact, according to Darriet [50], a new Olyset net reduces the entry rate of *An. gambiae* in the huts by 44% compared to an untreated net, whereas when it is 3 years old, its repellent effect is halved and this effect does no longer exist when it is washed. In addition, the dosage of the amount of insecticide carried out by Azondékon et al. [51] on LLINs fibers surface revealed a decrease in chemical efficacy only 6 months after the distribution of the LLINs Furthermore, the endophagic nature of *An. gambiae* in the study area is an asset for a preventive control based on IRS.

Regarding *An. funestus,* it represents the secondary vector encountered in the study area as it was found in a very low density compared to *An. gambiae.* This very low density has already been reported by Aikpon et al. [48] in the region of Atacora and by Gnanguenon et al. [47] in the Alibori (particularly in Kandi). The low abundance of *An. funestus* in Kandi and Gogounou may be due to the absence of its typical larval habitat (permanent or semi-permanent shaded freshwater streams, swamps, ponds and lakes) in these areas in view of the length of the drought period.

However, Aikpon et al. [52] reported a marked seasonal trend of *An. funestus* in Copargo (Donga region) with high abundance in the dry season. Conversely, the relatively high density of *An. funestus* in Djougou district, compared to other districts, is due to the existence of a small semi-permanent river, with surrounding vegetation, located not far from one of the study sites (Barienou).

The proportion of *An. gambiae* tested positive to circumsporozoitic antigen of *P. falciparum* was very high in the Alibori and in the Donga (SI = 8.05%), which stresses the need for the implementation of an effective malaria vectors control strategy in these two regions. A similar SI was previously reported in the Atacora, a northern region in Benin (SI = 6.63%) [48], in the Ouidah-Kpomasse-Tori region (southern Benin) (SI = 9.63%) [53] and in western Kenya (SI = 8.2%) [54]. This SI is lower than those observed in Eastern Gambia (SI = 17.73%) [55] and in Guinea-Bissau (SI = 12%) [56, 57]. This relatively high infection rate could be due to an increase in human–vector contact facilitated by anthropogenic behaviours (late hours at which people go to bed, non-usage of LLINs and others) and some factors that affect physical integrity (usage of sharp objects and lighted candles) and chemical effectiveness (high washing frequency) of LLINs. Other environmental factors, including high ambient temperature, prompt people to sleep outside without any protection against mosquito bites. This observation underscores the need to support IRS campaign with appropriate information, education and communication campaigns to combat this sleeping are misbehaviour in sprayed areas.

Although the infectivity rate was not the same in the four districts the highest rates were obtained in Djougou (16.84 infectious bites/man/month) and Copargo (17.64 infectious bites/man/month) and the lowest in Kandi (10.74 infectious bites/man/month) and Gogounou (11.04 infectious bites/man/month). These results suggest that the intensity of malaria transmission is higher in Donga region than in Alibori. Considering that the entomological inoculation rate is calculated using human biting and SI and, on the other hand, the similarity of SI in the two regions, it is legitimate to infer that the biting rate of *An. gambiae* was the main factor causing the difference observed between malaria transmission levels of the two regions. This deduction confirms the findings of Garrett-Jones [58], who reported that vector abundance is an important determinant in the malaria transmission level. In the case of the present study, the highest biting frequency of *An. gambiae* was observed in Donga region compared to Alibori (p < 0.0001), which could be due to some environmental characteristics (rainfall and soil humidity higher in the Donga region than in that of Alibori, thus promoting vector proliferation). Variation in malaria transmission levels between the two investigated regions may also be related to differences in topographies. In Alibori region, Kandi and Gogounou districts are located in areas of sloping plateaus that favour the runoff of water towards the south of the country after the rains. This situation prevents the on-site formation of a large number of breeding sites, resulting in lower vector abundance in Alibori region compared to Donga region. These results are consistent with findings of Omukunda et al. [59] who investigated similar bioecological areas in western Kenya.

The higher infection rates in rural areas compared to urban areas (p = 0) in the four districts confirm the spatial polymorphism in malaria epidemiology as previously observed by Sovi et al. [60] in the region of the Plateau, southeast of Benin. High EIRs obtained in rural areas were likely due to a high biting rate of *An. gambiae* resulting from an exponential proliferation in breeding sites meeting optimum conditions for development, in contrast to urban areas where the larval habitats are generally quite polluted and, therefore, more conducive to the development of *Culicines* [61]. Similar results have been observed in Dar es Salaam, Tanzania [62], in Ouagadougou, Burkina Faso [63], in Tori-bossito, southern Benin [64], in Kandi, northeastern Benin [31] and in the north–south transect of Benin [47]. In the study area, the risk of malaria infection was very high during the rainy season, but too low during the dry season despite the high parity rate in *An. gambiae* during this period compared to the rainy season. The period of malaria transmission is relatively longer in Donga region than in Alibori with a peak, respectively in August and October. These results are typical of tropical facies to which belong the two regions and characterized by a seasonal transmission of malaria interrupted by a dry season covering a non-negligible time in a year. A similar epidemiological facies was found in Zimbabwe, Kenya, Tanzania, northern areas of Nigeria, Benin, Ghana, Côte d’Ivoire and Guinea [65]. This observation should be taken into account to schedule spraying operations for a better impact of the intervention. Indeed, it is more appropriate to start the IRS campaign before the transmission period. In the four district covered by this study, June appeared to be the best period to start the IRS intervention. Since the CS formulation of pirimiphos methyl to be used for the next IRS campaign has a 4-month persistence period in field conditions of Atacora region [8], the short duration of malaria transmission in the Alibori region could be considered as an advantage.

## Conclusion

The Alibori and Donga regions are characterized by one transmission season relatively longer in Donga region than in Alibori. Spatio-temporal variation in entomological inoculation rates was also observed with higher rates in rural areas and during the rainy season. Given the duration of persistence of pirimiphos methyl selected for the IRS operations, the month of June would be the ideal period to start the implementation of the intervention. The information collected in this study provides a reference for the monitoring and evaluation of the IRS intervention in four districts of the study area.

## Abbreviations

EIR: entomological inoculation rate
HBR: human biting rate
HLC: human landing catch
SI: sporozoite index
IRS: indoor residual spraying
ITN: insecticide treated net
LLIN: long lasting insecticidal nets
PSC: pyrethrum spray catch
CS: micro-encapsulated formulation
